# What Does It Take for Research to Be Rehabilitation Research?

**DOI:** 10.3389/fresc.2022.823159

**Published:** 2022-07-11

**Authors:** Lena Aadal, Tove Lise Nielsen, Anders Bonde Jensen, Merete Bjerrum, Claus Vinther Nielsen, Sanne Angel

**Affiliations:** ^1^Hammel Neurorehabilitation and Research Center, Aarhus University, Aarhus, Denmark; ^2^Department of Clinical Medicine, Aarhus University, Aarhus, Denmark; ^3^Department of Occupational Therapy, Research Centre for Health and Welfare Technology, VIA University College, Aarhus, Denmark; ^4^Department of Oncology, Aarhus University Hospital, Aarhus, Denmark; ^5^Department of Public Health, Aarhus University, Aarhus, Denmark; ^6^Department of Clinical Medicine, The Centre of Clinical Guidelines–Danish National Clearing House, Aalborg University, Aalborg, Denmark; ^7^DEFACTUM, Central Denmark Region, Aarhus University, Aarhus, Denmark; ^8^Department of Clinical Social Medicine and Rehabilitation, Gødstrup Hospital, Central Denmark Region, Aarhus University, Aarhus, Denmark; ^9^Molde University College, Molde, Norway

**Keywords:** nominal group process, recommendations, rehabilitation, research, ICF

## Abstract

Six recommendations to facilitate rehabilitation research and supplement existing research practices were identified. Rehabilitation practice requires research addressing different long-term multi-faceted needs and perspectives of end users, including service users, professionals, politicians, and administrators. Research in rehabilitation should therefore integrate different research traditions and methods. Rehabilitation research with a broad focus is sparse, and most of the research takes its starting point in the biomedical research tradition. Through a nominal group process, we developed recommendations to emphasize important issues in rehabilitation research.

## Introduction

A growing recognition of the potential of rehabilitation has led to higher prioritization and acknowledgment of the need for high quality rehabilitation research (1, 2). Rehabilitation is, in essence, composed of a number of complex interventions (3)changing along the disease and recovery stages and involving shifting professionals. Thus, it is pivotal that rehabilitation research mirrors this complexity.

The benefits of a biopsychosocial approach in rehabilitation to reduce negative consequences of health issues has attracted growing attention and recognition (4). An increasing number of people need rehabilitation due to better medical treatment and demographic changes (5, 6). Worldwide, more than 2.4 billion people would benefit from rehabilitation (7). It has been established that rehabilitation may enhance self-efficacy and autonomy among people with disabilities (8) leading to decreasing health care needs. Consequently, rehabilitation may increase quality of life (QOL) and reduce the burden on health care systems and societies (5, 8).

According to WHO, rehabilitation addresses different aspects of the lived life and is “a set of interventions designed to optimize functioning and reduce disability in individuals with health conditions in interaction with their environment” (7). Thus, rehabilitation ranges from simple training that supports the individuals (here referred to as service users) to resume previous levels of functioning, to complex interventions for service users with multimorbidity and permanent loss of functioning that negatively affect several aspects of their everyday lives. The simultaneous parallel and linear interventions may take place over time and be considered as a continuous process involving physical, psychological and social aspects, especially for service users with limited resources, complex problems, and evolving needs. These aspects are reflected in the biopsychosocial understanding of the International Classification of Functioning, Disability and Health (ICF model) (9) and in established definitions of rehabilitation (7, 10). If rehabilitation is intended to improve functioning and facilitate recovery toward better QOL, autonomy and a meaningful life, then targeted coherent efforts are required, based on the service users' perspectives and life situations. Rehabilitation should thus be seen as a continuous collaborative process between the actively involved service user and an interdisciplinary group of social, vocational and healthcare professionals (11, 12). This complexity of independent variables and individualized approaches happening at different times makes 'rehabilitation research' complex and may explain why rehabilitation efforts are challenged by a limited knowledge base (2, 8). For example, in patients with low back pain, lack of rehabilitation research that covers broad aspects of functioning, QOL, autonomy and a meaningful life has been claimed (13). Similar problems have been described in stroke rehabilitation research (14). The lacking knowledge has implications for how rehabilitation research must be planned, executed and evaluated, if rehabilitation as evidence-based practice shall improve functioning, personal independence and a meaningful life. Thus, rehabilitation research must mirror the full rehabilitation process. Additionally, societal needs to optimize health-related costs imply that rehabilitation research should examine and test the effect of rehabilitation efforts as well as the individual service users' experiences of how the efforts contribute to increase and maintain functioning, QOL and autonomy. Complex rehabilitation interventions call for application of multiple research methods and designs from different scientific traditions to cover wider perspectives than traditionally explored by the randomized controlled trial (RCT) (2, 15). Moreover, long-term studies of multifaceted interventions are needed to examine all elements of a specific rehabilitation trajectory (2). Therefore, it becomes the responsibility of rehabilitation researchers to consider, optimally in collaboration with the service users, how rehabilitation research best meets the needs of the end users including service users, professionals, politicians, and administrators. The aim of the present paper is to offer recommendations for the broad field of rehabilitation research emphasizing the special characteristics of complex rehabilitation interventions that researchers should use to supplement established generic health-related research practices (16).

## Method

The Nominal Group Technique (NGT) was used to identify recommendations for rehabilitation research practice. This method is designed to explore opinions, generate ideas, and determine priorities in e.g., the health sector (17–20). Our group process followed the five steps process: (1): Introduction and explanation; (2): Silent generation of ideas building on individuals' knowledge, perspectives, and experiences; (3): Sharing individual contributions (round robin); (4): Group discussion for clarification; and (5): Prioritization of ideas (individual ranking).

The take-off of the nominal group process was a collaboration initiative between research, education and clinical practice in Central Denmark Region with focus on rehabilitation development in the region (21). Discussions were initiated at two meetings attended by a group of two representatives for service-users and 33 experts in rehabilitation (the expert group) from four independent sectors: hospitals; health and social care services; university, and university college. The expert group represented different backgrounds in relation to gender, education, role as clinician or researcher or patients' representative, organizational affiliation, and research tradition. From this expert group, six researchers with health and social care professional backgrounds representing the direct users were appointed to a working group to formulate targeted recommendations for rehabilitation research. The working group met four times from May to October 2019 to form the recommendations and at additional meetings to describe the final outcomes. The entire working group took part in all the five steps described below.

### Introduction and Explanation

The nominal group-process was initiated through the chairperson's presentation of the framework for rehabilitation, research obligations, and existing research within the field. Common challenges to rehabilitation research addressed at the inaugural meetings were presented.

### Silent Generation of Ideas. Individuals' Knowledge, Perspectives, and Experiences

Participants were asked to individually consider what they perceived as appropriate and high-quality rehabilitation research. All ideas were documented and accessible to all group members.

### Sharing Individual Contributions (Round Robin)

To elaborate and clarify the ideas, each member presented their ideas to the group, who posed in-depth questions.

### Group Discussion for Clarification

Structured discussion of all ideas from the common document. Large variations were identified, explained by the participants' different theoretical and scientific foundations. In-depth discussions led to a common understanding and nuancing of possible ideas related to complexity and processes in rehabilitation (19).

### Prioritization of Ideas

In this step, our process deviated from the NGT, as we omitted individual ranking of the generated ideas; instead, ideas were discussed until reaching consensus. This process led to a synthesis of recommendations of special importance within rehabilitation research. It became clear that some of the identified recommendations coincided with established, generic research practice; they were therefore deemed unnecessary to include. To avoid group processes and dominance influencing recommendations, and to consult the end users, the recommendations were subsequently discussed and amended at an expert group meeting also involving the service users.

## Results

The NGT process resulted in six recommendations for rehabilitation research as presented in Table 1 and explained in detail below.

**Table 1:** Recommendations for rehabilitation research.

1. Service users should be involved in the research process. In both the planning phase and during the research process, involvement of service users should reflect their illness experience, values, and knowledge of the rehabilitation efforts. 2. Rehabilitation research should have an explicit biopsychosocial perspective. Functioning should be assessed in interaction with health status, disease, and individual life circumstances. 3. Rehabilitation research should reflect the entire rehabilitation process. Rehabilitation research should reflect that rehabilitation is often a cross-sectional process of simultaneous and sequential multi-professional efforts. 4. Relevant knowledge gaps should be prioritized in collaboration with end users. It should be clarified if identified knowledge gaps are *relevant* to investigate, and which knowledge gaps are the most *pressing* to investigate according to both end users (service users, professionals, politicians, or administrators) and researchers. 5. Rehabilitation research should encompass considerations about implementation. Implications for the individual end user as well as for the competencies and organization of professionals should be considered. 6. Rehabilitation research should encompass considerations about how to disseminate results. Dissemination should reach the large number and variety of end-users and include implications for practice, research and education.

### Service Users Should Be Involved in the Research Process

In a number of countries, service-user involvement is required or recommended according to national health policy and legislation and is expected to improve healthcare-services (22–24). It secures democratic representation and empowerment of disadvantaged groups and presumably increases the quality and integration of research in clinical practice (24, 25). Two approaches for service-user involvement have been identified: The managerialist/consumerist approach aiming to “improve the product” and the democratic approach linked to organizations and movements. Both approaches strive to increase service-user influence in healthcare organizations and institutions, enabling them to gain better control over own lives (26, 27). Patient- and public involvement (PPI) in research is research carried out *with or by* the public (including service users) who act as participants, rather than research *on* the public/service users as subjects (28). The purpose with service user involvement is to ask relevant research questions about relevant issues. The extent of public involvement in rehabilitation research ranges from consultation to collaboration and co-research depending on question, perspective, and design (29).

### Rehabilitation Research Should Have an Explicit Biopsychosocial Perspective

Knowledge from different research areas is required and should be collected and analyzed from a holistic perspective (30). The ICF-model has proven to be a suitable framework for applying the holistic perspective to rehabilitation efforts (9), and an ICF matrix has been established to address this perspective in rehabilitation research. Based on the ICF-model, the rationale is that research is needed to inform how to improve physical functioning, activity, and participation of the person in interplay with the personal and environmental factors. The idea is that this contributes to being explicitly aware of the interacting components when long-term multifaceted interventions are investigated. In planning new studies, the ICF matrix can transparently structure the initial literature search and its results to clarify the existing knowledge base and pinpoint where new knowledge is warranted. Furthermore, the matrix can be useful in considering the impact of changes in one aspect of the ICF framework on other aspects of the framework. Table 2 shows the ICF matrix with the y-axis indicating the focus of research within five components of the ICF model and the x-axis provides space for research results (meaning or effect) in relation to each component. The ICF factor, health condition, is not included in the matrix, as the primary aim of rehabilitation is to address the close interaction between factors within and around the person that impair the person's everyday functioning and quality of life.

**Table 2:** ICF-matrix to provide an overview of research contributions and gaps.

	Body	Activity	Participation	Personal factors	Environmental factors	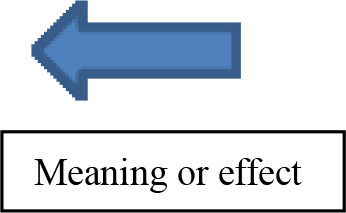
Body						
Activity						
Participation						
Personal factors						
Environmental factors						

*
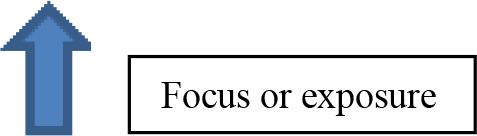
*

### Rehabilitation Research Should Reflect the Entire Rehabilitation Process

The research focus should adapt to ongoing changes in service users' needs due to their limited resources, complex and evolving problems (31). Research programs with longitudinal studies may be considered, as it is difficult to cover the entire rehabilitation process in one rehabilitation study. A series of studies focusing on different aspects of the rehabilitation-process and including patient reported outcomes could be a solution and requires a clarification of where in the rehabilitation process each study should be performed and why. Likewise, the necessity of investigating the cross-sectoral processes and efforts of the involved professions should also be acknowledged in rehabilitation research. It is pertinent to consider short- and long-term outcomes, as the latter can reveal developments or problems after termination of the active rehabilitation period.

### Relevant Knowledge Gaps Should Be Prioritized in Collaboration With End Users

There are many suggestions concerning how prioritizations in research should be established (32). It is important to acknowledge the mutual interest for evidence among end-users and researchers, but also their differing perspectives and priorities. It is therefore recommended to conduct an iterative process based on an actual problem. In researching e.g., low back pain, a problem could be identified by researchers, a group of service users, stakeholders or professionals. Next, relevant stakeholders should be involved to qualify and delineate the problem, for instance by using the ICF-matrix described above. Finally, existing knowledge is reviewed and incorporated to avoid reproduction of existing evidence, to identify knowledge gaps (using the ICF-matrix) and to prioritize the most relevant problem to investigate.

### Rehabilitation Research Should Encompass Considerations About Implementation

The gap between research-based knowledge and daily practice is well-known (33). Despite strategies for efficient transfer of new research-based knowledge into daily practice, it has been experienced that implementation of research into clinical practice may take a decade, and changes can be difficult to maintain (34, 35). A smoother transfer of new knowledge may be accomplished by addressing issues of relevance to the end users (26). This implies that a research protocol must specify the relevance of the project to the current practice in the field. Rehabilitation is a complex intervention involving multiple professionals and other stakeholders, and research is often aimed at informing the development of new tools, interventions or organizational approaches. Therefore, it is important that the protocol explicitly describes the necessary professional competencies and organizational structures required for implementation. If these prerequisites are not (yet) met in practice, suggestions for knowledge-translation must be articulated to ease the implementability of the results. Many granting bodies now expect such clearly articulated knowledge translation plans. An important way of ensuring knowledge translation is by prioritizing implementation studies: in line with the Medical Research Council's guidance for developing and evaluating complex interventions (3), we stress that implementation questions should be considered throughout the intervention development, feasibility testing, process, and outcome evaluation.

### Rehabilitation Research Should Encompass Considerations About How to Disseminate Results

All rehabilitation research should identify what the results mean for service-users, front line service providers, rehabilitation programs and policy makers so the implications and results are there for everyone to see. To reach the large number and variety of end-users of rehabilitation research, targeted presentations must be prioritized for narrower groups e.g., service user organizations and profession-, practice-, and sector-specific stakeholders. Qualitative findings have the potential to be more accessible to end-users than complex statistics (36), and a narrative format is a powerful tool to communicate scientific messages especially to non-academic audiences (e.g., the general public and policy makers) (37). Rehabilitation research, whether it has developed new insights, new tools, interventions, organizational approaches to rehabilitation etc., should always be followed by considerations and discussions of how the findings may guide future rehabilitation development and practice and facilitate further research and education.

## Discussion

In this paper, we have raised the need for targeted recommendations in rehabilitation research and have attempted to establish such recommendations to potentially clarify and increase the quality of the research and to ensure a transparent process from idea to implementation of results.

Rehabilitation is recognized as an important part of health care services. However, there is a need for more high-quality research, that encompasses a broad field of disciplines and methodologies covering the full spectrum from basic to applied science, and involves many different specialists with different research traditions. Ideally, a scientific process uses the most appropriate design to answer or illuminate the research question, and no single research tradition or method can be recommended or stand alone in rehabilitation research. The randomized controlled trial (RCT), often the default choice for intervention studies, has been claimed unfeasible for some clinical questions (e.g., if a particular presentation or condition is heterogeneous or rare) (38). The WHO guideline from 2017 stressed the limitations of randomized controlled trials and suggested that results from case-, observational or longitudinal studies can capture how environmental factors impact interventions at health system-level) (39). Likewise other types of evidence, as qualitative studies, are needed, too. Other methodological questions to address in relation to effect studies have been pointed out in relation to Cochrane studies. They relate to heterogeneous patient populations, complex rehabilitation interventions that are difficult to standardize, and to often vaguely described control conditions (38). The challenge of applying appropriate designs implies a need for future scholarly work on design development for rehabilitation research.

Using our recommendations can be a way to establish common ground for future discussion and development of holistic rehabilitation research, e.g., by using the biopsychosocial perspective of the ICF and the new ICF-matrix and the perspectives of the people receiving rehabilitation services to define the specific area of interest in each research project. It is important to emphasize that these recommendations are intended to guide researchers, decision-makers and funders within rehabilitation and rehabilitation research, all of whom do not necessarily have long experience with the discipline.

Although the recommendations are targeting rehabilitation researchers, there should be no doubt that we find it absolutely crucial that end users, including service users and stake holders, participate in the rehabilitation research process. End users are to be involved at distinct stages of the process: (1) priority setting and formulating research questions (2) study design, data collection and analysis (3) dissemination of findings and knowledge translation. This will improve the relevance and quality of rehabilitation research. Thus, the ultimate aim of the recommendations is to increase the knowledge base for rehabilitation by improving the actuality, relevance and implementability of rehabilitation research. Therefore, an important part of the recommendations stresses the involvement of all end users in the field of rehabilitation, in line with Solvang et al.'s emphasis on identifying and involving all agents in the field of rehabilitation at micro, meso, and macro level (40).

### Methodological Considerations

The NGT has been used in several fields including multidisciplinary health care integrating a patient-centered approach (20, 41, 42). Methodological rigor was optimized following the stepwise approach and recommendations for the NGT-technique (19). However, using the NGT raises some critical issues concerning the prioritization of the question in focus, participants' expertise, facilitators' competencies, group dynamics and equal discussions (19, 41, 42). The need for addressing specific recommendations for rehabilitation research was identified by 33 rehabilitation experts, and six experienced researchers from this group were appointed to fulfill the work. The process facilitator, an experienced group leader and expert in rehabilitation, secured balanced discussions (19, 20). The recommendations have been discussed by the 33 rehabilitation experts, including service users, who found them of high relevance and importance. However, the NGT application is a versatile exploratory method (42), and future use of the recommendations will examine their value in relation to further development of the field of rehabilitation research.

Rehabilitation can be defined both as a health strategy and a set of interventions based on the biopsychosocial model of functioning and disability (43). The International Society of Physical and Rehabilitation Medicine acknowledge this complexity and has developed categories linking different levels of healthcare in rehabilitation with areas of the scientific field to illustrate the diversity of research perspectives and related methodologies (44). This complexity is captured in our list of compiled and collectively presented principles that serve to support rehabilitation research.

## Data Availability Statement

The original contributions presented in the study are included in the article/supplementary material, further inquiries can be directed to the corresponding author.

## Author Contributions

All authors listed have made a substantial, direct, and intellectual contribution to the work and approved it for publication.

## Conflict of Interest

The authors declare that the research was conducted in the absence of any commercial or financial relationships that could be construed as a potential conflict of interest.

## Publisher's Note

All claims expressed in this article are solely those of the authors and do not necessarily represent those of their affiliated organizations, or those of the publisher, the editors and the reviewers. Any product that may be evaluated in this article, or claim that may be made by its manufacturer, is not guaranteed or endorsed by the publisher.
